# Management of peri-ovarian hematoma following oocyte retrieval in a pooling cycle IVF: Case report

**DOI:** 10.5935/1518-0557.20210079

**Published:** 2022

**Authors:** Garima Sachdeva, Devi R, Kamini A Rao, Madhuri Vidyashankar P

**Affiliations:** 1 Department of Reproductive Medicine, Milann, Bangalore, India

**Keywords:** IVF, hematoma, OPU, ultrasound, laparoscopy

## Abstract

Here we report a case of peri-ovarian hematoma following ovum pick-up in a patient in a pooling cycle IVF (in vitro fertilization). We have attempted to discuss the possible mechanisms for the development of hematoma in such patients, the common clinical presentation, monitoring, and management of these cases. The decision to operate or to manage conservatively forms an important aspect of managing such patients. This case report can help to keep the clinicians alert while managing this subgroup of patients.

## INTRODUCTION

Transvaginal ultrasound-guided oocyte retrieval is the safest and most widely used method of ovum pick-up (OPU) worldwide. Bleeding post-OPU is very rare, with a reported prevalence of vaginal bleeding and intra-abdominal/intraperitoneal bleeding post pick up of around 0.01% and 0.23%, respectively (Levi-Setti *et al*., 2018).

Here we discuss a case of peri-ovarian hematoma post-OPU, which required laparoscopy due to failed conservative management, with progressively increasing hematoma size and fall in hemoglobin levels.

## CASE REPORT

A 34-year-old woman with a history of primary infertility of 7 years visited our infertility center. She had a history of tuberculosis, for which anti-tuberculosis treatment (AKT4) was given. Diagnostic hystero-laparoscopy and chromopertubation were suggestive of bilateral patent tubes. Endometrial biopsy was positive for mycobacterium tuberculosis (TB-PCR) and she was treated again with AKT4.

The decision for in vitro fertilization (IVF) was made because of multiple failed TI/IUI cycles, high FSH (10.49 IU/ml), low AMH (0.41 ng/ml), and male factor (teratozoospermia and high DNA fragmentation index of 25%). The first OPU was done in 2019 using a mild stimulation protocol which was uncomplicated and 2- good quality blastocysts were formed.

The second ovum pick-up was planned after 1 month. We used a mild stimulation protocol (Letrozole and HMG 150 IU). The right ovary did not respond to stimulation. We retrieved two oocytes from the left ovary using a double lumen needle and only a single puncture was performed and two flushes each of 2 ccs were done for each follicle.

**Figure 1:**
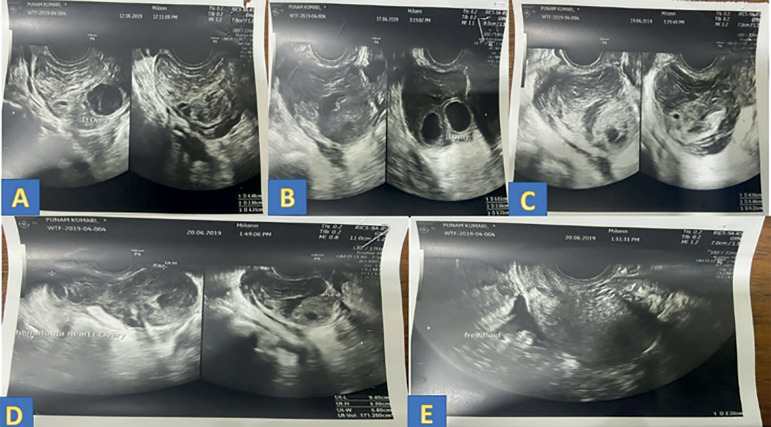
Post Pick left peri-ovarian hematoma. A- Day 3 post pick up- Left peri-ovarian hematoma measuring 4.48 cm X 4.31 cm X 2.86 cm B- Day 8 post pick up- Left peri-ovarian hematoma measuring 5.3 cm X 5 cm X 3.5 cm C- Day 10 post pick up- Left peri-ovarian hematoma measuring 6.2 cm X 5.4 cm X 4.5 cm D- Day 11 post pick up- Left peri-ovarian hematoma measuring 6.2 cm X 5.4 cm X 4.5 cm E- Day 11 post pick up- hemorrhagic free fluid in around left ovary (maximum vertical pocket-2.3 cm)

On day 3 post pick up, the patient developed complaints of mild abdominal pain. Her vitals were stable and her abdomen was soft. Ultrasound revealed a left ovarian hematoma 4.48 cm X 4.31 cm X 2.86 cm and hemorrhagic free fluid around the left ovary with a maximum vertical pocket- 2.6 cm (Figure 1A). Her right ovary measured 2.3 X 1.5 X 2 cm and the left ovary measured 4.4 X 3.7 X 3 cm. Two corpora lutea noted in the left ovary and its vascularity was well maintained. Her hemoglobin was 10 mg/dl, which was similar to her baseline hemoglobin. Her renal function test (creatinine-0.6 mg/dl), bleeding time (2 min 30 sec), clotting time (5 minutes), and urine culture were within normal limits.

On day 8, post-pick-up the intensity of abdominal pain increased and the patient developed tenesmus and pain during micturition. Upon examination, she was afebrile, she had a tenderness in the left iliac fossa, but no guarding or rigidity were present. She was hemodynamically stable. Her ultrasound scan revealed an increase in the size of the hematoma (5.3 cm X 5 cm X 3.5 cm) (Figure 1B) and a slight drop in hemoglobin (9.7mg/dl). There was neutrophilia (70%) and the patient was started on intravenous antibiotics. Daily monitoring of vitals, ultrasound findings, and alternate-day blood counts was done, and intravenous antibiotics were continued.

Day 11 post-pick-up, an ultrasound revealed an increase in the hematoma size (9.4 cm X 6 cm X 5.7 cm) (Figure 1D) and a further drop in hemoglobin (8.7 mg/dl). Table 1 shows changes in the ultrasound and clinical parameters post-pick-up. The decision for laparoscopy was made because of it.

**Table 1.** Changes in the ultrasound and clinical parameters post-pick-up.

POST OVUM PICK UP DAY	Ultrasound findings	Free Fluid (echogenic)Maximum Vertical Pocket (MVP)	Counts			
	Left peri-Ovarian Hematoma	Free Fluid (echogenic) Maximum Vertical Pocket (MVP)	Hemoglobin (g%)	Platelet Count (100.000/µl)	Total cells/µl	Differential Neutrophil -N Lymphocytes -L
DAY 3	4.48 X 4.31 X 2.86 cm	MVP-2.6 cm	10	1.4	10100	N70 L24
DAY 8	5.3 X 5.0 X 3.5 cm	MVP-2.6 cm	9.7	1.4	10300	N70 L24
DAY 10	6.2 X 5.4 X 4.5 cm	MVP-1.6 cm	8.9	1.4	8100	N7 L24
DAY 11	9.4 X 6.0 X 5.7 cm	MVP-2.3 cm	8.7	1.5	7600	N70 L21

**Figure 2:**
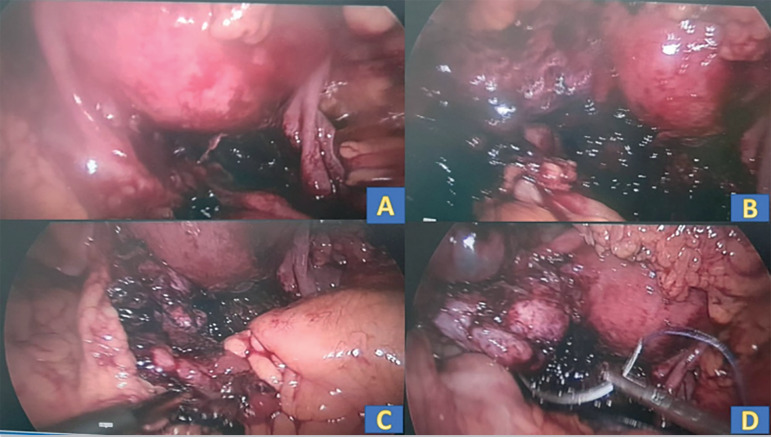
Intra-operative laparoscopy findings. **2A, 2B, 2C**- Organized clot and hemoperitoneum noted at laparoscopy **2D**- blood trickle from the left ovarian surface, hemostatic sutures taken

**Figure 3:**
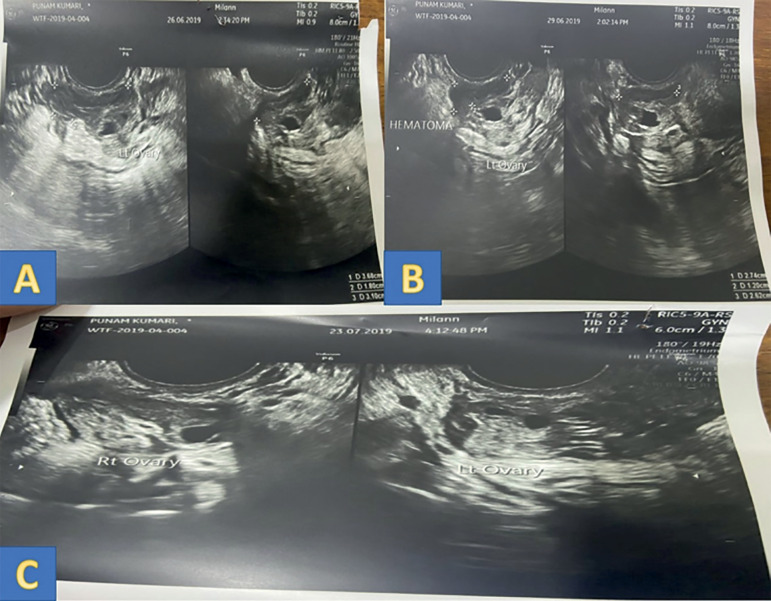
Post laparoscopy changes in the left peri-ovarian hematoma. **A- Day 6 post lap-** Left peri-ovarian hematoma measuring 3.11 cm X 2.97 cm X 1.80 cm **B- Day 10 post lap-** Left peri-ovarian hematoma measuring 2.7 cm X 2.6 cm X 1.20 cm **C-Day 28 post lap-** No hematoma

On laparoscopy hemoperitoneum of 100cc was noted, which was evacuated. An organized clot of 10 x 8 cm was noted around the left ovary extending to the pouch of Douglas and right ovary, also evacuated. On evacuating the clot, a bleeder was identified at the left ovarian puncture site; hemostatic sutures were taken (Figure 2D). Figure 2 demonstrates intraoperative laparoscopy findings. The patient was treated with a course of oral antibiotics and oral hematinics. The patient's symptoms improved in the postoperative period. There was a progressive decrease in the size of the hematoma and it almost disappeared on day 28 post-laparoscopy. Table 2 and Figure 3 demonstrate ultrasonographic and hematological findings in the postoperative period.

**Table 2.** Ultrasonographic and hematological findings in the post-laparoscopic period.

POST OVUM PICK UP DAY	Ultrasound findings	Free Fluid (echogenic)Maximum Vertical Pocket (MVP)	Counts			
	Left peri-Ovarian Hematoma	Free Fluid (FF)	Hemoglobin (g%)	Platelet Count (100.000/µl)	Total cells/µl	Differential Neutrophil -N Lymphocytes -L
DAY 3	3.68 X 3.10 X 1.80 cm	NO FF	10	1.8	8100	N60 L30
DAY 16	3.11 X 2.97 X 1.805 cm	NO FF				
DAY 10	2.7 X 2.6 X 1.20 cm	NO FF	10.4	2.82	4100	N58 L28
DAY 16	2.0 X 1.1 X 1.20 cm	NO FF				
DAY 28	NO HEMATOMA	NO FF				

## DISCUSSION

This patient presented with abdominal pain, tenesmus, and pain during micturition. Her symptoms were not correlating with the pick-up day findings, which triggered us to do an urgent scan, which revealed a peri-ovarian hematoma.

The various proposed mechanisms for intra-abdominal hematoma post-OPU include multiple punctures of the vaginal vault and the ovaries. The enlarged overstimulated ovaries especially in the lean PCOS (polycystic ovarian syndrome) patients proved to be a higher risk (Liberty *et al*., 2010). The other possible reasons include deranged coagulation profile, low platelet count, patient on any anticoagulants or on antiplatelets for some medical reason (Nouri *et al*., 2014). Rupture of the endometriotic cysts or hemorrhagic cysts is another possible cause (Lee *et al*., 2017). The previous history of pelvic tuberculosis, pelvic inflammatory disease, previous abdominopelvic surgeries, or previous OPU can result in neovascularization, and these fragile vessels can rupture resulting in hematoma in a subsequent pick up (Zhen *et al*., 2010).

The possible reasons for the peri-ovarian hematoma in this patient could be recent OPU, and history of tuberculosis, which could have resulted in neo-vascularization, and rupture of these fragile vessels might have resulted in the hematoma. In addition, the short duration between two cycles of pick-up could have added to this complication.

The Conservative approach is the first-line management in a hemodynamically stable patient as it saves the patient from another surgery or need for oophorectomy (Nouri *et al*., 2014). However, while doing so, one should keep a stringent watch on the vitals, symptoms, hematoma size, hemoglobin, and blood counts. Moreover, one should not postpone the surgery for too long if the patient is not improving as it can lead to life-threatening conditions like hemorrhagic shock, disseminated intravascular coagulation, and sudden death (Liberty *et al*., 2010). In this case, once we realized that the patient is not responding and there is a progressive increase in the hematoma size with a drop-in hemoglobin, the decision for surgery was made. Since the patient was hemodynamically stable, laparoscopy was performed instead of open surgery.

## CONCLUSION

Ovarian hematoma post-OPU is a rare complication. Post-pick-up abdominal pain, tenesmus, and pain during micturition can be important indicators of a pelvic hematoma. Previous OPU and tuberculosis may result in neovascularization and these fragile vessels can rupture during subsequent OPU resulting in intra-abdominal bleeding. A Conservative approach with close monitoring forms the first-line management in hemodynamically stable patients. However, if the patient does not improve or worsens with conservative management, surgery becomes imminent to prevent catastrophic complications.
